# Immunopeptidomics: Reading the Immune Signal That Defines Self From Nonself

**DOI:** 10.1016/j.mcpro.2022.100234

**Published:** 2022-05-11

**Authors:** Pierre Thibault, Claude Perreault

**Affiliations:** 1 Institute for Research in Immunology and Cancer, Université de Montréal, Montreal, Quebec, Canada; 2 Department of Chemistry, Université de Montréal, Montreal, Quebec, Canada; 3 Department of Medicine, Université de Montréal, Montreal, Quebec, Canada

Fifty years have passed since the study of Benacerraf and McDevitt (1) describing the exquisite regulation of the immune response by the major histocompatibility complex (MHC). The landmark discovery that T-cell activation requires corecognition of peptide antigens and self-MHC molecules (2) revealed not only a unique receptor–ligand interaction but also a delicate balance between autoimmune response and effective protection against infection. These seminal studies laid the foundation to our current understanding of how immune cells distinguish between self and nonself.

T cells display a remarkable selectivity and discriminate self and nonself from an astonishing number of antigen peptides presented by MHC molecules, a property that is largely defined during their development in the thymus. Furthermore, the MHC locus is the most polymorphic region of the human genome, and this structural diversity enables MHC molecules to present a wide distribution of peptide antigens originating from the degradation of endogenous (MHC class I) or exogenous (MHC class II) proteins. The biogenesis of peptide antigens is an intricate process shaped not only by environmental stimuli but also by specific proteolytic enzymes located in distinct organelles. The antigen repertoire, collectively referred to as the immunopeptidome, cannot be inferred from transcript or protein abundance and mainly relies on mass spectrometry analyses of peptides eluted from MHC complex. However, mass spectrometry alone is not sufficient to unveil the full repertoire of antigens concealed by MHC molecules. Indeed, structural variants arising from genomic polymorphisms or noncanonical regions of the genome are absent from the reference proteomes, and their discovery requires more elaborated databases that leverage proteogenomic search strategies (3). Recent technological advances in MHC isolation methods, mass spectrometry sensitivity, and novel database search approaches have fueled immunopeptidomic discoveries and open up new perspectives for cancer immunotherapy. It is in this context that we assembled experts and opinion leaders in immunopeptidomics to contribute to this special issue and share their findings and views of this blossoming field of research.

A perspective article by Jonathan Yewdell (4) takes us on an historical journey of the immunopeptidome from the discovery of antigens presented by MHC to its future outlook in tumor immunology. All nucleated cells express MHC I molecules that present not only normal and abnormal self-antigens but also nonself pathogens to the effector CD8+ T cells. Defective ribosomal products account for a major source of misfolded nascent proteins that are subsequently degraded, thus contributing to the pool of peptides available to MHC I molecules. Defective ribosomal products have been implicated in early alert of the immune system about impending infections, and work from Arie Admon (5) suggests that the corresponding proteins represent a sizeable proportion of MHC I peptides upon cell stimulation with type I and II interferons. Immunopeptidomic studies have also enabled the identification of modified and noncanonical peptides. The discovery of proteasome-catalyzed peptide splicing has raised important questions regarding the actual proportion of these peptides in the immunopeptidome, and Arie Admon (6) and Michele Mishto (7) are providing opposing views on the occurrence of these events. A review by Mahoney *et al.* (8) discusses the occurrence of phosphorylated antigens in cancer and viral infections with a particular emphasis on dysregulation of p53, pRb, and PP2A.

Macrophages, dendritic cells, and B cells express MHC II molecules and typically present abnormal or nonself pathogen antigens for the activation of CD4+ T cells. In many tumor types including glioblastoma, MHC II complexes are often not expressed. Work from Forlani *et al.* (9) revealed the expression of MHC II peptides following the stable expression of the MHC II transactivator in glioblastoma cell lines and highlight their potential as peptide vaccines. Demers *et al.* (10) used B cells to show the intrinsic responses and adaptations in antigen presentation after heat shock without infection and found that upregulation of costimulatory molecules and MHC II proteins specifically reshaped the MHC II immunopeptidome and primed B cells for an immune response. Antigen presentation in MHC II complexes depends on its peptide-loading catalyst (human leukocyte antigen [HLA]-DM) and its associated modulator (HLA-DO) though this interaction is still incompletely understood. By using single MHC II allele antigen-presenting cells, Olsson *et al.* (11) described how the ratio of HLA-DM and HLA-DO affects MHC II peptide presentation. Taylor *et al.* (12) reviewed MHC II immunopeptidomics and discussed current challenges in immunoaffinity enrichment, improvement in peptide identification and epitope prediction, and the potential of MHC II peptides as targets for future immunotherapies.

Immunopeptidomic workflows typically use different mass spectrometry acquisition methods, search engines, and allele prediction algorithms. While data-dependent acquisition is typically used in immunopeptidomic workflow, Pak *et al.* (13) implemented data-independent acquisition and assessed its sensitivity and accuracy by matching these data against libraries of growing complexity. Klaeger *et al.* (14) achieved deeper immunopeptidome coverage and sensitivity using microscale basic reverse-phase fractionation and ion mobility separation. A contribution by Pollock *et al.* (15) described a semiautomated workflow for the isolation and multiplex quantification of MHC I peptides and demonstrated the routine identification of >4000 unique antigens from 250 million cells. Parker *et al.* (16) provided a detailed analysis of the performance of four search engines commonly used in the field of immunopeptidomics and highlighted differences in sensitivity and bias for each. MHC-binding prediction tools are commonly used in immunopeptidomics to identify peptide antigens likely present in the study population. To improve allele prediction, Pyke *et al.* (17) developed a pan-allele MHC-binding algorithm (SHERPA) using 167 HLA alleles and obtained increased precision compared with competing algorithms. While a fix affinity threshold is generally applied across all alleles, work from Reardon *et al.* (18) found that allele-specific thresholds are preferable when analyzing a few alleles. A computational and analytical approach to discriminate true HLA ligands from coisolated HLA-independent proteolytic peptides is proposed by Fritsche *et al.* (19), where they described tools and highlighted how to circumvent false ligand identification. To view immunopeptidomic data, Sirois *et al.* (20) developed MhcVizPipe, a graphical user interface–based software tool for quality control in mass spectrometry–based immunopeptidomics.

Several contributions of this issue leveraged immunopeptidomic workflow to identify putative tumor-specific antigens. Qi *et al.* (21) described a proteogenomic approach to identify MHC I tumor-specific antigens in melanoma (“hot” tumor) and in an epidermal growth factor receptor mutant lung adenocarcinoma (“cold” tumor). MHC-I immunopeptidome analyses of murine fibrosarcoma following oncolytic reovirus and immune checkpoint blockade combination therapy identified a subset of peptide antigens that stimulate CD8 T-cell responses in treated animals (22). The combination of immunopeptidomics and RNA-Seq enabled the identification of tumor-specific antigens in an acute myeloid leukemia cell line THP-1 (23) and in colorectal cell lines and primary specimens (24) and revealed that a large proportion of these antigens derived from alternative reading frames or noncoding genomic regions.

Vaccines based on peptide antigens specific to neoplastic cells offer a glimmer of hope for the treatment of cancer. However, significant optimization is required in terms of antigen selection, modes of delivery, biomarker of drug efficacy, and combination therapy, before peptide vaccines can become an effective therapy. Nelde *et al.* (25) reviewed current approaches in the development of peptide vaccines and their practical implications as future therapeutics.

This special issue aims at taking the pulse of the evolving field of immunopeptidomics. Here, we attempted to feature many recent developments in immunopeptidomic workflows encompassing different enrichment and labeling strategies, mass spectrometry acquisition methods, sequencing, and allele prediction softwares. Although multiple challenges still exist in terms of standardization and sensitivity, we believe that continuing technological advancements in immunopeptidomics will provide new opportunities for basic and applied research in immunology. Such improvements represent a critical unmet need, particularly in immuno-oncology, where leveraging immunopeptidomic discoveries could improve the efficacy of current immunotherapies, including therapeutic vaccines and bispecific T-cell engagers.

## References

[1] Benacerraf B., McDevitt H.O. (1972). Histocompatibility-linked immune response genes. Science.

[2] Zinkernagel R.M., Doherty P.C. (1974). Restriction of *in vitro* T cell-mediated cytotoxicity in lymphocytic choriomeningitis within a syngeneic or semiallogeneic system. Nature.

[3] Nesvizhskii A.I. (2014). Proteogenomics: Concepts, applications and computational strategies. Nat. Methods.

[4] Yewdell J. (2022). MHC class I immunopeptidome: Past, present & future. Mol. Cell. Proteomics.

[5] Komov L., Melamed Kadosh D., Barnea E., Admon A. (2021). The effect of interferons on presentation of defective ribosomal products as HLA peptides. Mol. Cell. Proteomics.

[6] Admon A. (2021). Are there indeed spliced peptides in the immunopeptidome?. Mol. Cell. Proteomics.

[7] Mishto M. (2021). Commentary: Are there indeed spliced peptides in the immunopeptidome?. Mol. Cell. Proteomics.

[8] Mahoney K.E., Shabanowitz J., Hunt D.F. (2021). MHC phosphopeptides: Promising targets for immunotherapy of cancer and other chronic diseases. Mol. Cell. Proteomics.

[9] Forlani G., Michaux J., Pak H., Huber F., Marie Joseph E.L., Ramia E., Stevenson B.J., Linnebacher M., Accolla R.S., Bassani-Sternberg M. (2021). CIITA-transduced glioblastoma cells uncover a rich repertoire of clinically relevant tumor-associated HLA-II antigens. Mol. Cell. Proteomics.

[10] Demmers L.C., Wu W., Heck A.J.R. (2021). HLA class II presentation is specifically altered at elevated temperatures in the B-lymphoblastic cell line JY. Mol. Cell. Proteomics.

[11] Olsson N., Jiang W., Adler L.N., Mellins E.D., Elias J.E. (2022). Tuning DO:DM ratios modulates MHC class II immunopeptidomes. Mol. Cell. Proteomics.

[12] Taylor H.B., Klaeger S., Clauser K.R., Sarkizova S., Weingarten-Gabbay S., Graham D.B., Carr S.A., Abelin J.G. (2021). MS-based HLA-II peptidomics combined with multiomics will aid the development of future immunotherapies. Mol. Cell. Proteomics.

[13] Pak H., Michaux J., Huber F., Chong C., Stevenson B.J., Muller M., Coukos G., Bassani-Sternberg M. (2021). Sensitive immunopeptidomics by leveraging available large-scale multi-HLA spectral libraries, data-independent acquisition, and MS/MS prediction. Mol. Cell. Proteomics.

[14] Klaeger S., Apffel A., Clauser K.R., Sarkizova S., Oliveira G., Rachimi S., Le P.M., Tarren A., Chea V., Abelin J.G., Braun D.A., Ott P.A., Keshishian H., Hacohen N., Keskin D.B. (2021). Optimized liquid and gas phase fractionation increases HLA-peptidome coverage for primary cell and tissue samples. Mol. Cell. Proteomics.

[15] Pollock S.B., Rose C.M., Darwish M., Bouziat R., Delamarre L., Blanchette C., Lill J.R. (2021). Sensitive and quantitative detection of MHC-I displayed neoepitopes using a semiautomated workflow and TOMAHAQ mass spectrometry. Mol. Cell. Proteomics.

[16] Parker R., Tailor A., Peng X., Nicastri A., Zerweck J., Reimer U., Wenschuh H., Schnatbaum K., Ternette N. (2021). The choice of search engine affects sequencing depth and HLA class I allele-specific peptide repertoires. Mol. Cell. Proteomics.

[17] Pyke R.M., Mellacheruvu D., Dea S., Abbott C.W., Zhang S.V., Phillips N.A., Harris J., Bartha G., Desai S., McClory R., West J., Snyder M.P., Chen R., Boyle S.M. (2021). Precision neoantigen discovery using large-scale immunopeptidomes and composite modeling of MHC peptide presentation. Mol. Cell. Proteomics.

[18] Reardon B., Kosaloglu-Yalcin Z., Paul S., Peters B., Sette A. (2021). Allele-specific thresholds of eluted ligands for T-cell epitope prediction. Mol. Cell. Proteomics.

[19] Fritsche J., Kowalewski D.J., Backert L., Gwinner F., Dorner S., Priemer M., Tsou C.C., Hoffgaard F., Romer M., Schuster H., Schoor O., Weinschenk T. (2021). Pitfalls in HLA ligandomics-how to catch a li(e)gand. Mol. Cell. Proteomics.

[20] Kovalchik K.A., Ma Q., Wessling L., Saab F., Duquette J.D., Kubiniok P., Hamelin D.J., Faridi P., Li C., Purcell A.W., Jang A., Paramithiotis E., Tognetti M., Reiter L., Bruderer R. (2022). MhcVizPipe: A quality control software for rapid assessment of small- to large-scale immunopeptidome datasets. Mol. Cell. Proteomics.

[21] Qi Y.A., Maity T.K., Cultraro C.M., Misra V., Zhang X., Ade C., Gao S., Milewski D., Nguyen K.D., Ebrahimabadi M.H., Hanada K.I., Khan J., Sahinalp C., Yang J.C., Guha U. (2021). Proteogenomic analysis unveils the HLA class I-presented immunopeptidome in melanoma and EGFR-mutant lung adenocarcinoma. Mol. Cell. Proteomics.

[22] Kim Y., Konda P., Murphy J.P., Paulo J.A., Gygi S.P., Gujar S. (2022). Immune checkpoint blockade augments changes within oncolytic virus-induced cancer MHC-I peptidome, creating novel antitumor CD8 T cell reactivities. Mol. Cell. Proteomics.

[23] Scull K.E., Pandey K., Ramarathinam S.H., Purcell A.W. (2021). Immunopeptidogenomics: Harnessing RNA-seq to illuminate the dark immunopeptidome. Mol. Cell. Proteomics.

[24] Cleyle J., Hardy M.P., Minati R., Courcelles M., Durette C., Lanoix J., Laverdure J.P., Vincent K., Perreault C., Thibault P. (2022). Immunopeptidomic analyses of colorectal cancers with and without microsatellite instability. Mol. Cell. Proteomics.

[25] Nelde A., Rammensee H.G., Walz J.S. (2021). The peptide vaccine of the future. Mol. Cell. Proteomics.

